# N6-methyladenosine (m6A) modification and its clinical relevance in cognitive dysfunctions

**DOI:** 10.18632/aging.203457

**Published:** 2021-08-30

**Authors:** Bingying Du, Yanbo Zhang, Meng Liang, Zengkan Du, Haibo Li, Cunxiu Fan, Hailing Zhang, Yan Jiang, Xiaoying Bi

**Affiliations:** 1 Department of Neurology, Shanghai Changhai Hospital, The Second Military Medical University, Shanghai, PR China; 2 Department of Psychiatry, Faculty of Medicine and Dentistry, University of Alberta, Edmonton, AB, Canada; 3 Faculty of Basic Medical Sciences, The Second Military Medical University, Shanghai, PR China; 4 Department of Biochemistry and Cell Biology, Geisel School of Medicine, Dartmouth College, Hanover, NH 03755, USA; 5 Department of Oral and Maxillofacial-Head Neck Oncology, Shanghai Ninth People’s Hospital College of Stomatology, Shanghai Jiao Tong University School of Medicine, Shanghai, PR China

**Keywords:** m6A regulator, cognitive impairment, WGCNA, KEGG pathways, apolipoprotein E

## Abstract

Background: N6 adenosine methylation (m6A) is the most abundant internal RNA modification in eukaryotic cells. Dysregulation of m6A has been associated with the perturbations of cell proliferation and cell death in different diseases. However, the roles of m6A in the neurodegenerative process and cognitive dysfunction are unclear.

Methods: We systematically investigated the molecular alterations of m6A regulators and their clinical relevance with cognitive dysfunctions using published datasets of Alzheimer's Disease (AD), vascular dementia, and mild cognitive impairment (MCI).

Findings: The expressions of m6A regulators vary in different tissues and closely correlate with neurodegenerative pathways. We identified co-expressive m6A regulators SNRPG and SNRPD2 as potential biomarkers to predict transformation from MCI to AD. Moreover, we explored correlations between Apolipoprotein E4 and m6A methylations.

Interpretation: Collectively, these findings suggest that m6A methylations as potential biomarkers and therapeutic targets for cognitive dysfunction.

Funding: This work was supported by the National Natural Science Foundation of China (81871040) and the Shanghai Health System Talent Training Program (2018BR29).

## INTRODUCTION

Alzheimer’s disease (AD) and vascular dementia (VD) are common neurocognitive disorders [1–3]. The cerebrospinal fluid (CSF) concentrations of phosphorylated Tau 181 (Tau-181) and amyloid-beta 42 (Aß-42) are considered biomarkers for AD [4–6]. There are no diagnostic or therapeutic biomarkers for VD [7]. Mild cognitive impairment (MCI) is a transitional and reversible stage that can diverge to normal aging and neurocognitive disorder [8, 9]. MCI increases the risk of developing neurocognitive disorders [9], but the trajectory of individuals varies. Identifying biomarkers of neurocognitive disorders in the MCI stage is critical for early diagnosis and intervention [10].

With the advances in biochemistry and sequencing techniques, over 150 RNA modifications have been identified in the past decade [11, 12]. N6-methyladenosine (m6A) is the most common RNA modification in eukaryotic cells [13–17]. The abundance of m6A in the brain gradually increases with age and peaks in adulthood [18]. M6A is highly enriched in adult brain tissue [19, 20] and plays a critical role in neurogenesis, neurodevelopment, and neurological disorders [18, 20–23]. M6A modification on messenger RNA (mRNA) affects the proliferation and differentiation of neural progenitor cells [24–26], and elucidating dysregulations and alterations of m6A perturbations facilitates a comprehensive understanding of RNA methylation-based stem cell or gene-targeted diagnosis and therapy [17, 27].

**Figure 1:**
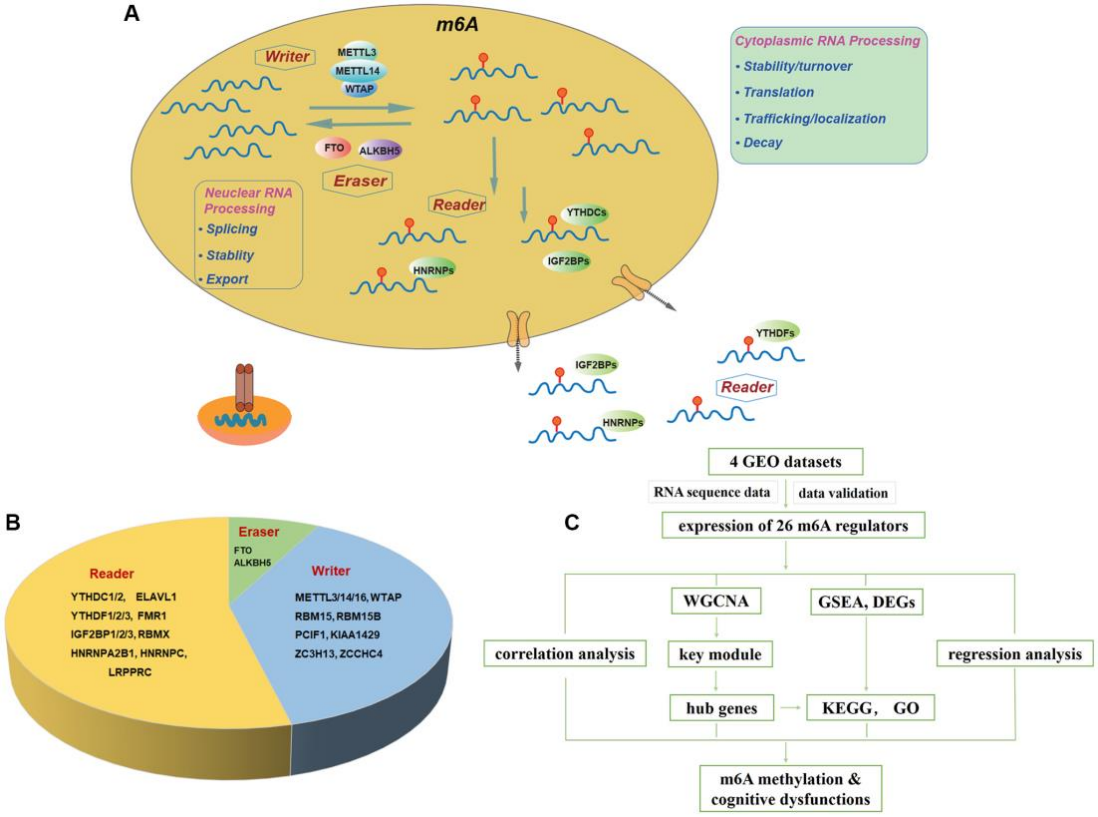
**Landscape of included m6A regulators.** (**A**) Overview of dynamic biological processes of m6A RNA methylation mediated by “writers”, “erasers” and “readers” in nucleus and cytoplasm. (**B**) Distribution of “writers”, “erasers” and “readers” among included 26 m6A regulators. (**C**) Workflow of the study design. Abbreviations: GEO: Gene Expression Omnibus; WGCNA: Weighted Gene Co-expression Network Analysis; GSEA: Gene Set Enrichment Analysis; DEGs: differentially expressed genes; KEGG: Kyoto Encyclopedia of Genes and Genomes; GO: Gene Ontology.

M6A modification is dynamically regulated by methyltransferases (also known as “writers”), demethylases (“erasers”) and binding proteins (“readers”) (Figure 1A, 1B) [15, 28]. This methylation installed by the “writers” can be reversed by “erasers” [29]. Dysregulations of m6A have been associated with the perturbations of cell proliferation and cell death in different diseases [11, 30, 31].

Alternations of RNA methylation modified genes in the central nervous system (CNS). Little evidence has elucidated the relationships between m6A regulators and neurodegeneration, such as dementia [32]. A recent study by Han et al. using APP/PS1 transgenic mice indicated that m6A abnormality (such as METTL3 and FTO genes) is closely related to AD [33]. To gain a thorough understanding regarding cognitive malfunction from a new perspective, we systematically investigated the molecular alterations of m6A regulators and their associations with AD, VD, and MCI using the database search.

## MATERIALS AND METHODS

### Collection of m6A regulators

Our study was designed and conducted according to the flow chart (Figure 1C). Briefly, twenty-six m6A regulators were selected accordingly to recent publications [15, 34–37], including ten writers (METTL3, METTL14, METTL16, RBM15, RBM15B, WTAP, KIAA1429, PCIF1, ZCCHC4, ZC3H13), two erasers (FTO, ALKBH5) and fourteen readers (YTHDC1, YTHDC2, YTHDF1, YTHDF2, YTHDF3, IGF2BP1, IGF2BP2, IGF2BP3, HNRNPA2, HNRNPC, FMR1, RBMX, LRPPRC, ELAVL1) (Figure 1B). Figure 1A summarized the landscape of related regulators, including classification, biological functions, and molecular mechanisms.

### Acquisition of microarray datasets and preprocessing

The gene-expression dataset with full clinical annotation was obtained from Gene Expression Omnibus (GEO), a publicly sponsored genomic database operated by the National Institutes of Health (NIH). GEO provides open access to many gene expression data from biological and statically comparable samples [38]. In total, four eligible datasets regarding AD, VD, or MCI, including GSE122063, GSE63060, GSE63061, and GSE84422, were selected. There are 711 blood samples from GSE63060 (*n* = 382) and GSE63061 (*n* = 329), including 238 control, 189 MCI and 284 AD. GSE122063 consists of 136 brain samples from either frontal or temporal lobe, and patients are divided into control (*n* = 11), VD (*n* = 8), and AD (*n* = 12) groups. GSE84422 collected 1053 post-mortem brain samples from 125 subjects with a full spectrum of AD.

The microarray platforms provided by Illumina and Agilent were downloaded in the format of normalized matrix files. The dataset retrieved from Affymetrix was downloaded in raw “CEL” form. The R/Bioconductor algorithm “RMA” and package “SVA” were used to preprocess gene chips normalization among datasets and to remove batch effects and other latent variations [39]. The overall workflow was presented in Figure 1C.

### Analysis of unsupervised clustering for m6A regulators

Unsupervised clustering analysis is an effective machine learning tool for exploring the patterns of datasets in a complex system, which has been applied to AD studies [40] and single-cell RNA sequencing applications [41]. In the current study, the m6A-related regulators were classified into several distinct endotypes by employing unsupervised clustering methods [42, 43] and m6A modification patterns based on the mRNA sequencing of 21 m6A regulators were hereafter determined for further research. Consensus Cluster Plus R package was conducted to perform 1000 times repetitions to guarantee the stability of classification [44]. The number of clusters was determined by the consensus clustering cumulative distribution function (CDF) result (Supplementary Figure 1). The purpose of the CDF plot is to find the *k* at which the distribution reaches an approximate maximum, which indicates maximum stability, and after which divisions are equivalent to random picks rather than the true cluster structure [44]. Besides, patients were classified into different groups for deeper analysis by adopting an unsupervised clustering method for analyzing the significant difference in different clusters by consensus clustering.

### Identification of differential gene expression

After data normalization, differentially expressed genes (DEGs) in datasets of GSE122063, GSE63060, and GSE63061 were identified using the “Limma” package from R/Bioconductor software [45]. The significance of DEGs was set as the adjusted *P*-value <0.05 and threshold of |log2FC|≥1. Different expression levels of 26 m6A regulators among groups were further verified by unsupervised clustering analysis.

### Exploring KEGG pathway enrichment

After identifying DEGs in frontal and temporal cortices, Database for Annotation, Visualization and Integrated Discovery (DAVID, https://david.ncifcrf.gov/, ver. 6.8) was further used to identify Kyoto Encyclopedia of Genes and Genomes (KEGG) pathway enrichment of DEGs in the above two brain regions. The cutoff criteria were set as *P* values of 0.05. The results of top10 KEGG pathways in both cortices were picked up and constructed in a bubble plot via R Studio.

### Gene set enrichment analysis and functional annotation

Gene set enrichment analysis (GSEA) has successfully been applied to interpret the molecular pathway activated in different biological states [46]. In this study, software “GSEA” (https://www.gsea-msigdb.org/) was utilized to identify the gene up- or down-regulation after filtering for gene set size (min = 5, max = 500) and ranked by *t*-score [47]. The gene sets of “c2.cp.kegg.v7.1.symbols” (MSigDB database) were used for GSEA analysis. The FDR-corrected *q*-value <0.25 and *P*-value <0.05 were set for significance.

### Weighted gene co-expression network analysis

Weighted gene co-expression network analysis (WGCNA) was used to extract highly correlated clinical traits and calculate module membership measures from the data sets [48]. “WGCNA” R package was applied to determine hub genes and clinical traits-related modules among microarrays [48]. Genes with variations in the top 25% were extracted from DEGs analysis. Biweight miscorrelation (corType = “Pearson”) was set to detect the outliers. The topological overlap matrix (TOM) was transformed to find the connectivity in the adjacent matrix. Genes were after that divided into multiple sensitive modules according to the TOM-based dissimilarity measurement. Other analysis setting regarding the identification of key modules included soft-threshold power = 7, scale free R2 = 0.9, height = 33, cut height = 0.2, and minimal module size = 10. Subsequently, genes from the highest correlated module were picked up to perform Gene Ontology (GO) and Kyoto Encyclopedia of Genes and Genomes (KEGG) pathway analyses. Hub genes were determined by defying gene significance (GS) >0.3 and module membership (MM) >0.8.

### Comparing the expression levels according to genotypes of apolipoprotein E gene

The association between apolipoprotein E (APOE) gene ɛ4 allele and m6A methylation regulators in AD patients was examined by extracting data from GSE29652. Eighteen post-mortem brain samples of AD were categorized into APOE ɛ4+ or APOE ɛ4− subtype. The expression levels of m6A regulators were compared between APOE ɛ4 genotype groups after data normalization and DEGs extraction.

### Statistical analysis

The Statistical Package for the Social Sciences (SPSS) version 24.0 was used for statistical analysis. Patients in GSE63060 and GSE63061 were sub-grouped by age according to their cognitive functions. Normal distributed continuous variables were described using mean ± SD; categorical variables were presented as percentages (%). Differences between groups were compared by *t*-test, one-way ANOVA, or Kruskal-Wallis test for continuous variables, and chi-square for categorical variables.

Correlation analyses were carried out to compute the strength of interrelationships between clinical traits and gene expression traits. Correlations between m6A regulators were computed by Spearman correlation analyses and visualized by the “corrplot” package in the R program. Univariate analysis examinations, filtering the meaningful independent variables, followed by multivariate logistic regression analysis, were conducted to estimate the association between m6A methylation levels and MCI and AD.

All statistical *P* values were two-tail, and *p* < 0.05 was regarded as statistically significant.

### Data sharing statement

All relevant data supporting the key findings are available from the corresponding author upon reasonable request.

## RESULTS

### Overview of included datasets and m6A regulators

Seven hundred and seventy-one blood samples from GSE63060 and GSE63061 were stratified into three age categories (≤70, 71–79, ≥80). There are significant differences in age distribution among CTL, MCI, and AD groups (*χ*^2^ = 26.2, *P* < 0.001) (Supplementary Table 1). 50% of patients over 80 years had AD, which is significantly higher than in younger age groups, supporting that AD is age-related.

### Expression patterns of m6A-related regulators vary with cognitive dysfunctions

Firstly, expression levels of m6A RNA methylation regulators from different samples in the same groups were compared. As we can see, nearly half of the proteins have various expressive phenotypes in the control group. The expression profile of YTHDC2 mainly was enriched in the frontal lobe, while RBMX and FTO were in the temporal lobe, and IGF2BP3 was in the blood (Supplementary Figure 2A). The expression profiles were different in AD patients, and even more prominent expressive differences were found. The expression levels of METTL3 and KIAA1429 were highest in the frontal lobe, while PCIF1 had the highest expression in the temporal lobe, and YTHDC2 had the highest expression in the blood (Supplementary Figure 2B). The combined results indicated that the modification phenotypes of m6A-related regulators varied in different samples.

**Figure 2:**
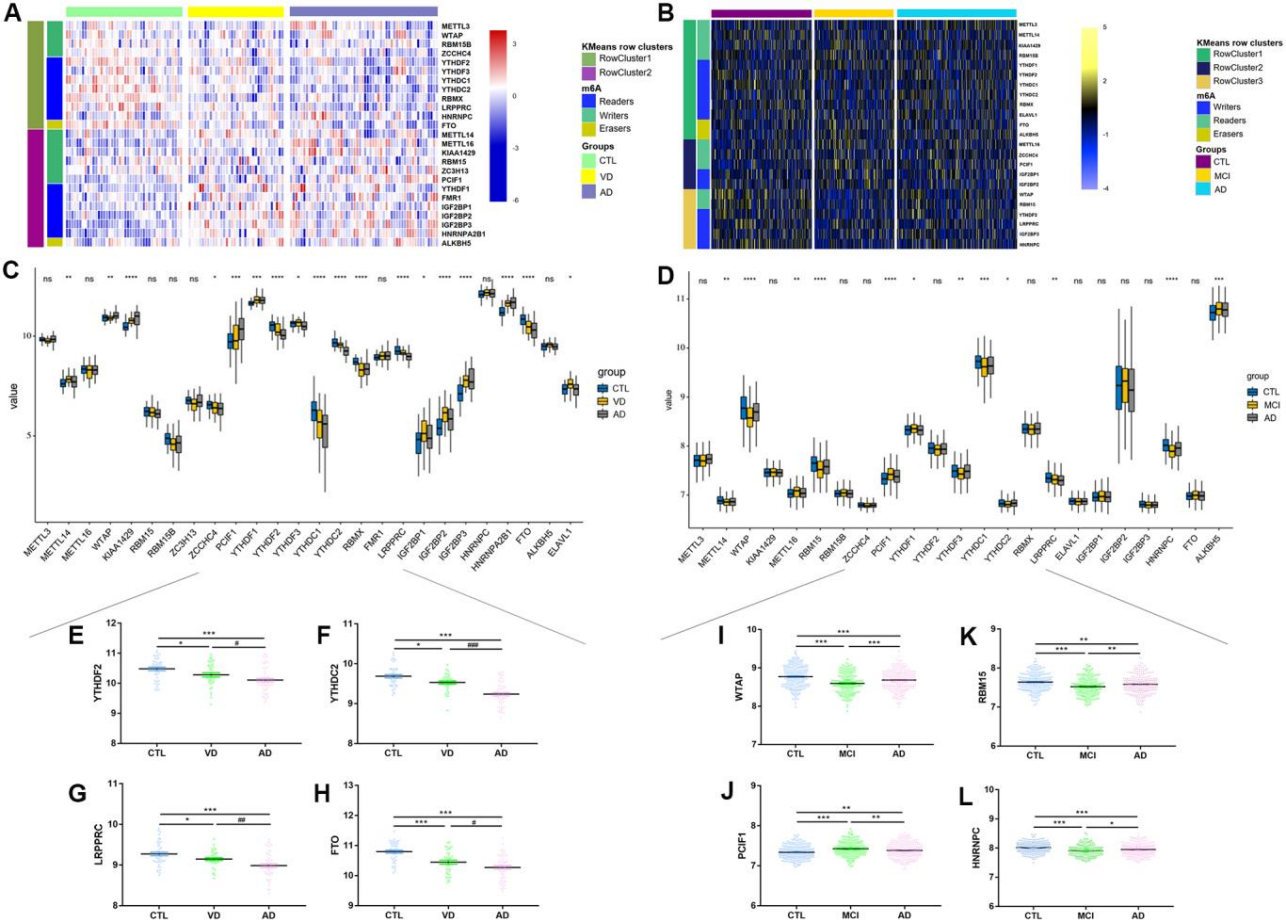
**Mutation frequency distribution of m6A regulators across different groups in brain and blood samples.** (**A**) Unsupervised clustering of 26 m6A regulators in GSE122063, annotated according to cognitive statues. Red represented high expression of regulators and blue represented low expression. (**B**) Unsupervised clustering of 26 m6A regulators in GSE63060 and GSE636061, annotated according to cognitive statues. Yellow represented high expression of regulators and blue represented low expression. (**C**–**D**) The expression profiles of 26 m6A regulators in brain and blood samples. (**E**–**L**) Box plots showing representative expression differences between CTL, VD and AD from brain sample, or between CTL, MCI and AD from blood sample. ^*^p < 0.05, ^**^p < 0.01, ^***^p < 0.001, ^****^p < 0.0001, ns: no significance.

To further explore these m6A regulators’ expression patterns, our attention was fixed on comparing different levels of cognitive functions within the same tissues. Unsupervised clustering analysis was performed by classifying into several clusters, according to K-means. When classified according to brain regions, two distinct clusters were identified. We noted that m6A Cluster1 was relatively enriched in the frontal lobe (Supplementary Figure 2C), with writers METTL3, METTL14, readers YTHDF2/3, and YTHDC1/2 had the most significant enrichment (Supplementary Figure 2D–2K). No noticeable enrichment difference was found by visual inspection in m6A Cluster2. When classified by cognitive status, the exhibition was different (Figure 2). We discovered that m6A Cluster1 was distinctively enriched in the CTL group, especially in readers and eraser FTO (Figure 2A, 2C). As for m6A Cluster2, it was mainly enriched in readers of VD and AD groups, with IGF2BP1/2/3 and HNRNPA2B1 were the most obvious (Figure 2A, 2C). The most significant divergently expressed regulators between the three groups were seen in Figure 2E–2H (YTHDF2, *F* = 10.612; YTHDC2, *F* = 36.231; LRPPRC, *F* = 13.354; FTO, *F* = 22.789). Besides, our previous research has found the differences in epigenetics modifications in diverse brain regions (unpublished), so when further comparisons were conducted based on brain domains, the expression imbalance was similar to the combined results (Supplementary Figure 2C).

There were three m6A Clusters, and 13 differently expressed regulators between CTL, MCI, and AD in blood samples (Figure 2B, 2D). Cluster 2 was mainly enriched in the VD group, Cluster 3 was mainly enriched in the CTL group, with WTAP (*F* = 22.354), RBM15 (*F* = 12.609), PCIF1 (*F* = 13.629), HNRNPC (*F* = 12.133) had the most distinctive mutations among groups (all Ps < 0.0001, Figure 2I–2L). Interestingly, we noticed that the expression tendencies of MCI were always different from those of CTL or AD groups (Figure 2D), indicating that expressional alterations of m6A regulators played a crucial role in mediating the progression of cognitive dysfunctions.

### Interactions between m6A-related regulators

Considerable evidence has proved that cross-talks of m6A regulators were ubiquitous [35, 49]. Therefore, we further investigated collaboration among writers, erasers, and readers by constructing protein-protein interaction (PPI) networks. In general, correlated expression patterns and genetic alterations were not only within the same biological regulators but also among writers-erasers-readers (Supplementary Figure 3). Except for recently reported proteins PCIF1, ZCCHC4, and LRPPRC, the other regulators' expressive patterns were significantly associated with each other. Mostly evident interactions were observed between proteins within the ten writers. Close interrelationships were also found between writers with readers or erasers. Relatively few interactions were existed between the 14 readers, while complicated interactions between erasers FTO and ALKBH5 were apparent in the PPI network (Supplementary Figure 2).

**Figure 3:**
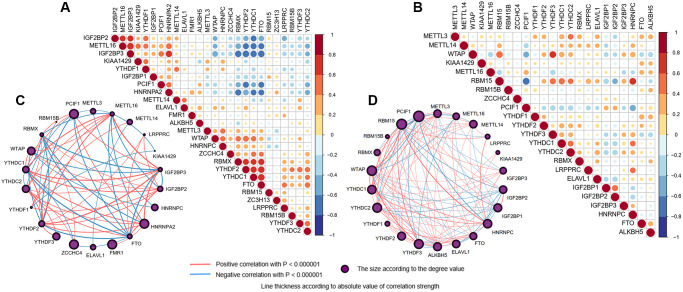
**Interaction among m6A RNA methylation regulators.** (**A**, **B**) Spearman correlation analysis of the 26 m6A methylation regulators in brain (**A**) and blood (**B**) samples. Positive correlation was marked with red and negative correlation with blue. (**C**, **D**) The interaction between the 26 m6A methylation regulators by constructing PPI network in brain (**C**) and blood (**D**) samples, respectively. The circle size was determined by the degree value. The lines linking regulators showed their interactions, and thickness showed the correlation strength between regulators. Positive correlation was marked with red and negative correlation with blue.

Correlations between m6A regulators in different samples and two brain cortices were computed by Spearman correlation analyses and visualized by the “corrplot” package in the R program. The lines linking regulators showed their interactions, and thickness showed the correlation/interference strength between regulators (Figure 3, Supplementary Figure 3). As we can see, the result of brain samples showed strong co-occurrences between eraser FTO with readers RBMX, YTHDF2, and YTHDC1. At the same time, negative correlations were exhibited with writers METTL16, IGF2BP3 and HNRNPA2, and PCIF1. The closest interference was found between METTL16 and IGF2BP3 (Figure 3A, 3C; R = 0.792, *p*-value < 0.0001). According to brain regions, we found more inseparable expression profiles (Supplementary Figure 4). For example, the absolute values of the correlation coefficient between IGF2BP3 with FTO and YTHDF2 were both more than 0.80 in the temporal lobe (Supplementary Figure 4B, 4D; R = −0.818 and −0.813, respectively, both Ps < 0.0001). While in the frontal lobe, the interaction strength between regulators was somewhat weaker, but the absolute values of the correlation coefficient between IGF2BP3 and YTHDF2, FTO and YTHDC1 were also close to 0.80 (Supplementary Figure 4A, 4C; R = 0.773 and −0.753, respectively, both Ps < 0.0001). Besides, the interactions between METTL16, TYHDF2, YTHDC1, and IGF2BP3 with other regulators were always the strongest, indicating intimidate connections among m6A-related regulators.

As for blood samples, we found that the overall correlations were less close to those in the brain. The writer RBM15 was positively correlated with writer WTAP and readers YTHDC1/2 and YTHDF3, while negatively correlated with writer PCIF1 and readers IGF2BP1/2, with the closest relationship existed within the same functional protein class between writers RBM15 and PCIF1 (Figure 3B, 3D; R = −0.638, *p*-value < 0.0001).

### Identification of m6A-related biological functions

**Figure 4:**
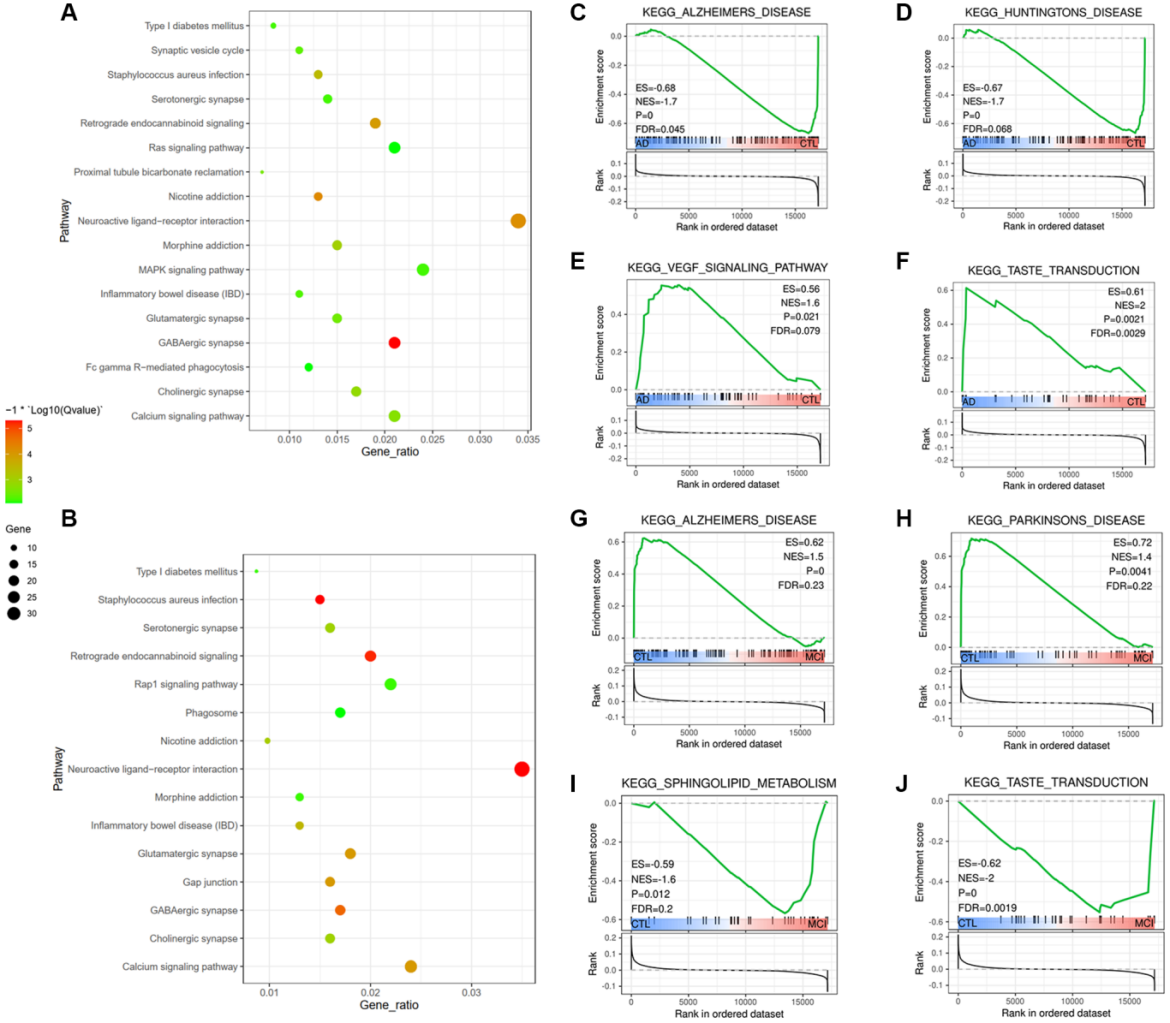
**Identification of differentially regulated molecular pathways and m6A-related biological functions.** (**A**, **B**) Functional annotation of the genes with different expression in frontal (**A**) and temporal (**B**) lobes using KEGG pathway. (**C**–**J**) Representative differentially regulated pathways are shown by analyzing blood samples. Pathways with increased activation included pathways indicative of Alzheimer's Disease (**C**) and VEGF signaling pathway (**E**), pathways with reduced activation included Huntington's Disease (**D**) and taste transduction (**F**) in AD group compared with CTL group; (**G**–**J**) Pathways with dysregulated activation in MCI group compared with CTL group.

Potential biological processes of m6A-related regulators were further investigated. 502 and 674 DEGs were identified in frontal and temporal cortices, respectively, and they were subsequently performed by the Kyoto Encyclopedia of Genes and Genomes (KEEG) pathway analysis. Bubble plots showed that DEGs in two lobes were both mainly enriched in neuro-modulatory activities, including neuroactive ligand-receptor interaction, serotonergic synapse, GABAergic synapse, glutamatergic synapse, and cholinergic synapse. Other pathways, such as the retrograde endocannabinoid signaling pathway and Calcium signaling pathway, were also mainly involved (Figure 4A, 4B). In addition, the Ras signaling pathway, MAPK signaling pathway were typically enriched in the frontal lobe (Figure 4A), while Rap1 signaling pathway, phagosome, and Gap junction were in the temporal lobe (Figure 4B), demonstrating that biological functions in various brain regions were similar in general but also slightly different.

### Estimation of differentially regulated molecular pathways

We used the Gene Set Enrichment Analysis (GSEA) algorithm to identify molecular pathways differentially regulated according to cognitive dysfunctions. Of the 65 pathways in the AD group compared to the CTL group, 41 pathways were activated, while 24 pathways were down-regulated (Additional file 1). Generally, GSEA-based analysis highlighted a broad dysregulation of genes related to neurodegenerative disorders. Among the latter are genesets associated with AD, Parkinson’s Disease and Huntington’s Disease, taste transduction, sphingolipid metabolism, and calcium signaling pathway (Figure 4C–4J). It is interesting to find that taste transduction was the only pathway mediated between AD and MCI, and it was up-regulated in AD vs. CTL. However, down-regulated both in MCI vs. CTL and AD vs. MCI (Figure 4J, 4F, Additional file 1), indicating that taste transduction malfunction might play a role in cognitive impairment progress. Besides, Pathways such as the VEGF signaling pathway, complement and coagulation cascades, JAK-STAT signaling pathway, and MAPK signaling pathways were activated in the AD group compared to the CTL group (Additional file 1). The identified dysregulated pathways and related genes, e.g., VEGF, JAK, MAPK, and complements, could be therapeutic targets of AD.

### Construction of gene co-expression network and module of interest identification

**Figure 5:**
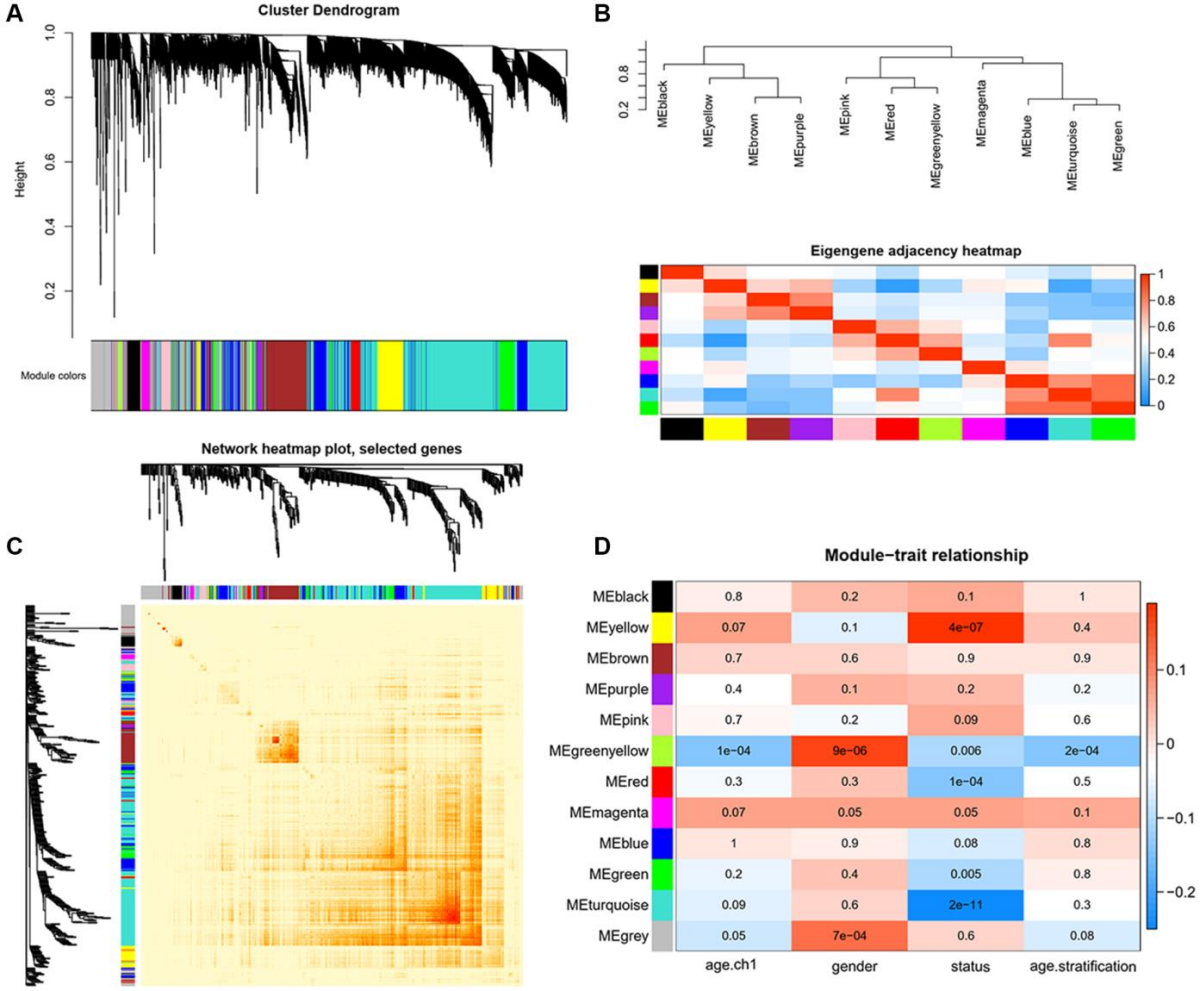
**Identification of key modules correlated with clinical traits by WGCNA.** (**A**) Cluster dendrograms of all genes, with dissimilarity based on topological overlap, and then various module colors were assigned. (**B**) The upper panel displays the hierarchical clustering dendrogram of hub genes that summarize the analyzed modules and branches of the dendrogram group with eigengenes are closely correlated. The lower panel shows the eigengene adjacency heatmap, with the trait weight included. The darker red color represents higher adjacency, while darker blue color represents low adjacency. (**C**) Heatmap plot of Topological Overlap Matrix (TOM) among selected genes. Each module corresponds to a branch in the hierarchical clustering dendrogram. Modules demonstrate more saturated yellow or even red colors indicate higher co-expression interconnection. Genes locate at the tip of each branch indicate highest interconnection with the rest of the genes in the module. (**D**) Heatmap of the associations between module eigengenes and clinical traits. Each row and column correspond to a module eigengene or a clinical trait. The plot is colored by corresponding correlation according to the legend, and each cell contains the corresponding *P*-value. The red color represents positive correlation, while blue color represents negative correlation.

In this study, weighted gene co-expression network analysis (WGCNA) [50] was performed to identify the key modules most associated with AD and MCI clinical traits. Ages were stratified into three subgroups: ≤70, 71–79, and ≥80 years old. After setting the soft threshold = 7, 6 outliers were removed (height = 33), and the left 705 genes were, after that, theoretically classified according to the expression pattern (Supplementary Figure 5). Firstly, we identified 11 modules of highly co-expressed genes by considering clinical traits, including age/age stratification, gender, cognitive status (Figure 5A). Eigengene adjacency heatmap was further conducted and revealed four main branches among the genes, which verified the above interconnections (Figure 5B). Unique color identifiers were assigned to each module, with gray represented the remaining poorly connected genes. Then, the co-expressed gene network was constructed, and the topological overlap matrix (TOM) heatmap plot was employed to show the network landscape (Figure 5C). The rows and columns in the TOM plot corresponded to various genes. The color intensity represented values of Pearson correlation coefficients, which meant that the higher color intensity indicates higher co-expression similarity between genes included in the network. Herein, genes appeared highly interconnected between module green with modules turquoise and blue, module purple with module brown by visual inspection (Figure 5C).

**Figure 6:**
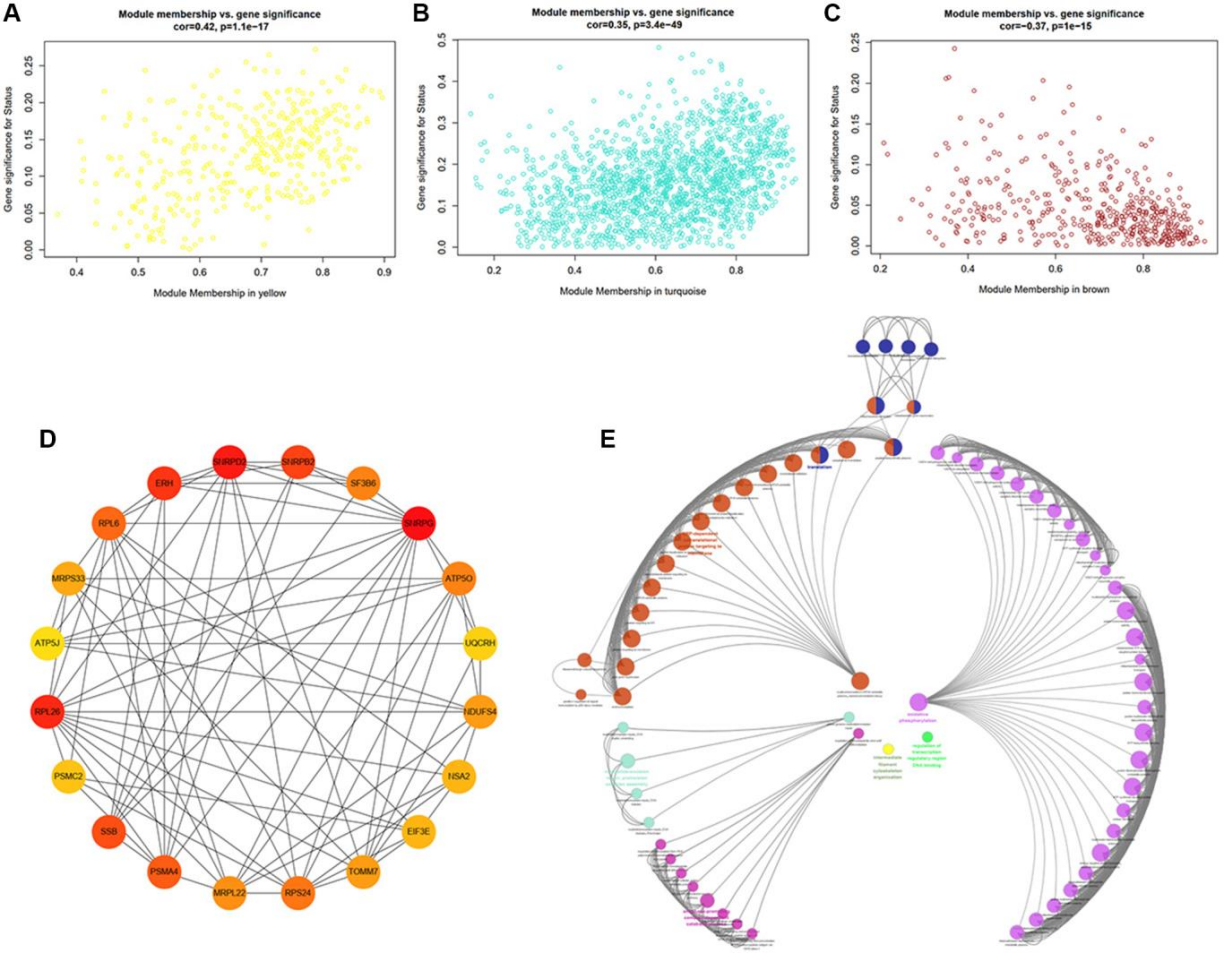
**Identification of hub genes and functional annotation of the WGCNA module highly correlated with clinical traits.** (**A**–**C**) Scatter plots of eigengenes in the representative modules yellow (**A**), turquoise (**B**), and brown (**C**), which have highly significant correlation between Gene Significance (GS) and Module Membership (MM). (**D**) PPI network of the top20 hub genes in the module turquoise. The circles with darker red represent higher gene rank. (**E**) Biological functional annotation of the top20 hub genes in the module turquoise by Geno Ontology (GO) enrichment analysis.

To further strengthen the study of crucial module identification, we defined a measurement to discern the statistical significance between modules with clinical traits. As depicted in the Heatmap, the associations between module eigengenes and clinical traits were colored by corresponding plot, with a darker color (red or blue) indicating strong correlations. Therefore, modules turquoise and yellow were closely intimated with cognitive statues (Figure 5D), indicating that genes in these two modules had high relationships with different cognitive performance levels. The investigation of gene significance (GS) was further performed to find the modules most biologically connected to clinical traits. (Figure 6A–6C). What is more, the existence of significant correlations between GS and module membership (MM) implied that genes within the module turquoise tended to strongly interrelate to cognition (correlation coefficient = 0.35, *P* = 3.4e-49; Figure 6B).

A total of 1666 genes in module turquoise and the top20 hub genes, including SNRPG, SNRPD2, RPL26, ERH, SNRPB2, and SSB, were selected, setting GS >0.3 and module membership (MM) >0.8. We can tell that these genes were inseparably associated with each other (Figure 6D). Herein, GO enrichment analysis was performed to identify potential biological functions of module turquoise-related genes (Figure 6E). The result revealed that genes within the module turquoise were most significantly enriched in translation, peptide biosynthetic process, nuclear-transcribed mRNA catabolic process, mRNA/RNA catabolic process.

### Prediction of associations between m6A-related methylations and cognitive dysfunctions

In order to assess the effect of m6A-related regulators on the prevalence of cognitive dysfunctions, unconditional logistic regressions were used. As expected, age predicts AD (OR = 1.055, 95%CI 1.025–1.087) and MCI (OR = 1.056, 95%CI 1.022–1.091), while gender has no prediction values. WTAP, ZCCH4, and HNRNPC from blood samples were found to have protective roles in MCI prevalence (Supplementary Table 2-1). While AD, METTL3, YTHDF3, and ALKBH5 were predicted to significantly increase the odds ratios, with YTHDF3 having the most apparent effect (OR = 8.033, 95%CI 2.047–31.523). On the contrary, METTL14, WTAP, YTHDC1, IGF2BP2, HNRNPC, and FTO had a protective effect on AD (Supplementary Table 2-2). When we further analyzed the relationships between MCI and AD, we surprisingly found that males with MCI had prone to deteriorating into AD compared to females (OR = 1.490, 95%CI 1.003–2.213), while age failed to predict the outcome. Besides, METTL3, WTAP, and RBM15 increased the possibility of occurrence, with METTL3 had the most apparent effect (OR = 6.984, 95%CI 1.778–27.432), while YTHDF1, YTHDC1, and LRPPRC were just the opposite (Supplementary Table 2-3).

Further analysis from brain regions of frontal and temporal lobes demonstrated age (OR = 1.067, 95%CI 1.050–1.085) and IGF2BP2 (OR = 2.633, 95%CI 1.248–5.552) were promotive to AD, while METTL16 and LRPPRC were protective to the prevalence. The model’s overall predictive value, sensitivity, and specificity were 79.4%, 70.8%, and 80.0%, respectively.

### Exploration of correlations between APOE ɛ4 and m6A-related methylations

**Figure 7:**
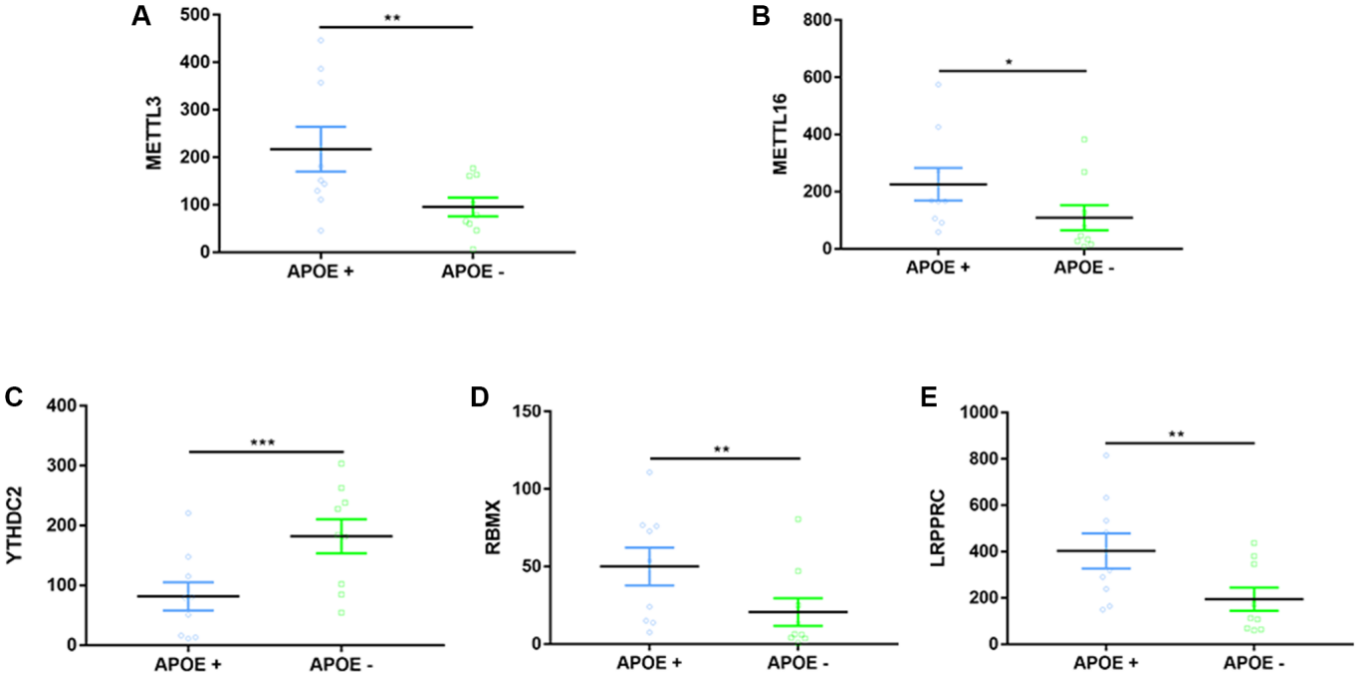
**Expression differences on m6A-related regulators between APOE ɛ4+ and APOE ɛ4− groups.** (**A**–**E**) Five representative DEGs between two groups. ^*^p < 0.05, ^**^p < 0.01, ^***^p < 0.001.

The ɛ4 allele of the human apolipoprotein E gene (APOE) is a well-proven genetic risk factor for the late-onset form of AD [51]. Studies have proved that the ɛ4 allele of APOE was differentially methylated in AD [52, 53]. However, whether RNA methylation changes the presence of the APOE ɛ4 allele is still unknown. Thus, data from GSE29652, which contains 18 postmortem brain samples, were extracted and analyzed according to APOE ɛ4+/−. As a result, five DEGs were dug out (Figure 7). Except for YTHDC2 was downregulated in the ɛ4+ group, the other four DEGs (METTL3, METTL16, RBMX, and LRPPRC) were upregulated epigenetic alterations of RNA methylation might be related to APOE ɛ4 dysfunction in AD (Figure 7A–7E).

## DISCUSSION

The discovery of m6A mRNA methylation has extended a new dimension in post-transcriptional gene expression [29]. Various animal experiments have previously suggested the modulation roles of m6A on neuronal functions [19, 20, 54–57]. However, the relevance of m6A RNA methylation in cognitive dysfunction remains mostly unexplored. The current study has identified certain m6A-related regulators and related modification patterns, which might serve as novel biomarkers and therapeutic strategies for cognitive dysfunction.

Firstly, we found that m6A-related regulators’ expression differs in different tissues and different cognition levels. A little evidence has confirmed that m6A regulators were closely related to hippocampal-dependent learning and memory [22, 56] and further proved the inner regulating mechanisms [58, 59]. We found that FTO, YTHDC2, and YTHDF2 were the most divergently expressed regulators in the brain between different cognitive groups. However, different from the above findings, we found that expression of METTL3 had no significant difference between CTL, MCI, and AD, while METTL14 had the highest expression in CTL but lowest in MCI. This may partly explain that the above two studies were based on animal research and tissue from the hippocampus, while we analyzed samples of blood from the elderly [22, 56]. Besides, recently reported studies have proved that oligodendrocyte (OL) lineage progression was accompanied by changes along with m6A-related regulators, such as METTL14, YTHDF2, or even a novel reader PRRC2A [60, 61]. Considering our previous findings that OLs and myelin were closely associated with animal cognition [62, 63], we may uncover a m6A-specific cognition modification. Conditional inactivation of m6A components might result in decreased OLs numbers, and CNS hypomyelination and the latter have been implicated in the development of cognitive impairment. Together, we hypothesize that it is possible to regulate OLs proliferation and differentiation by modulating RNA methylation and improving hippocampus-dependent cognitive function. Further *in vivo* and *in vitro* experiments are needed to validate the conjecture.

KEGG analysis revealed that DEGs in the brain were significantly enriched in neuroactive ligand-receptor interaction, consistent with a previous study. They reported that the regulation of neuroactive ligand-receptor interaction associated with AD was not preserved in healthy and MCI networks [64]. Another multinational study confirmed that genes regarding neuroactive ligand-receptor interaction were closely related to memory-modulation [65]. As for DEGs in blood, GSEA results surprisingly revealed enrichment of taste transduction. The alterations of taste perception were commonly found in aging and neurodegenerative disorders [66–68]. For example, frontotemporal dementia (FTD) is characterized by alterations in gustation, eating behaviors [69, 70], and appetite alteration are also significantly found in AD [70, 71]. The type 1 taste receptor member 3 (T1R3) is closely involved in taste perception and highly abundant in cognition-related brain areas, such as the hippocampus and cortex [72]. Besides, bioinformatics tools confirmed that the T1R3 receptor processes a strong structural similarity with metabotropic glutamate receptors, and the latter is crucial for learning, memory, and behavior [73, 74]. The loss of the T1R3 subunit is thereby demonstrated to cause learning and memory impairment [68]. Taken together with our and others' data suggest that taste transduction plays a crucial role in cognition procession, and alterations of taste might implicate as an indicator of cognitive dysfunction.

The present study has dug out critical proteins associated with MCI and AD through WGCNA. Belonging to the small nuclear ribonucleoprotein peptide family, SNRPG has been identified as one of the bridge regulators in the module network closely connected to MCI and AD [75, 76]. The decreased expression level of SNRPG might participate in the progression from MCI to AD [76]. Meanwhile, SNRPD2 interacts with nuclear retention elements, and a decrease of SNRPD2 also correlates with pathogenesis from MCI to AD [76, 77]. Moreover, several genes show overlaps in the potential pathogenesis of cancers. It is consistent with a previous bioinformatic study regarding VD by our group [accepted but unpublished] and another PD-associated research [78], which collectively indicated that genes associated with neurodegenerative diseases were always abnormally dysregulated in cancers [76]. Therefore, we may conclude that our study contributed to a better understanding of the pathological mechanisms from MCI to AD. Proteins like SNRPG, SNRPD2, or even cancer-related are expected to be novel biomarkers to predict for patients with MCI who are more likely to progress to AD.

For the first time, we demonstrated in the present study that APOE ɛ4 is closely correlated to five RNA methylation regulators (METTL3, METTL16, YTHDC2, RBMX, LRPPRC) in the AD brain. The ε4 allele of APOE is the most common and influential genetic risk factor for developing AD [79]. Lee et al. once reported that expression of all APOE RNA species was significantly higher in the AD brain than those in the control brain [80]. Similarly, we found a significant increase in most m6A-related regulators within the AD APOE ɛ4+ group, suggesting a complex regulation of epigenetic alterations between the ɛ4 allele and AD. A prospective cohort study by Keller et al. once reported an interaction between FTO and APOE. It proved that those carrying genes of both FTO and AOPE ɛ4 had an increased risk for dementia [81]. They further figured out that FTO's effect on dementia or AD risk mainly was through interaction with the APOE ɛ4 allele [81]. We did not find the difference of FTO expression between APOE ɛ4+/− groups, suggesting that we adopted different samples (brain vs. blood). Consistent with Han's study reporting an elevated level of METTL3 in AD mice, we found that the AD APOE ɛ4 + group has a higher expression of METTL3 [33]. Ectopic expression of RBMX was reported to decrease the APOE receptor’s splicing and was critical to cholesterol homeostasis and, possibly, AD development [82]. Loss or mutation of LRPPRC may contribute to manifestations of neurofibromatosis type 1, which has characteristics of cognitive dysfunction [83]. No previous studies have ever reported relationships between METTL16, YTHDC2 with AOPE ɛ4, or cognition.

## CONCLUSIONS

The current study has demonstrated the prevalent genetic and expression alterations of RNA methylation regulators according to cognitive impairment. These differently modified patterns of m6A regulators deserve to be highlighted because they are tightly correlated with cognitive malfunctions. The systematic evaluation of m6A regulators-related molecular alterations might lay a critical foundation for understanding the characteristics of cognition. It will also contribute to guiding more therapeutic strategies for dementia.

## Supplementary Materials

Supplementary Figures

Supplementary Tables

Additional file 1

## References

[1] Kivipelto M, Mangialasche F, Ngandu T. Lifestyle interventions to prevent cognitive impairment, dementia and Alzheimer disease. Nat Rev Neurol. 2018; 14:653–66. 10.1038/s41582-018-0070-3 30291317

[2] Shigemizu D, Akiyama S, Asanomi Y, Boroevich KA, Sharma A, Tsunoda T, Matsukuma K, Ichikawa M, Sudo H, Takizawa S, Sakurai T, Ozaki K, Ochiya T, Niida S. Risk prediction models for dementia constructed by supervised principal component analysis using miRNA expression data. Commun Biol. 2019; 2:77. 10.1038/s42003-019-0324-7 30820472 PMC6389908

[3] Robinson L, Tang E, Taylor JP. Dementia: timely diagnosis and early intervention. BMJ. 2015; 350:h3029. 10.1136/bmj.h3029 26079686 PMC4468575

[4] Olsson B, Lautner R, Andreasson U, Öhrfelt A, Portelius E, Bjerke M, Hölttä M, Rosén C, Olsson C, Strobel G, Wu E, Dakin K, Petzold M, et al. CSF and blood biomarkers for the diagnosis of Alzheimer's disease: a systematic review and meta-analysis. Lancet Neurol. 2016; 15:673–84. 10.1016/S1474-4422(16)00070-3 27068280

[5] Fagan AM, Shaw LM, Xiong C, Vanderstichele H, Mintun MA, Trojanowski JQ, Coart E, Morris JC, Holtzman DM. Comparison of analytical platforms for cerebrospinal fluid measures of β-amyloid 1–42, total tau, and p-tau181 for identifying Alzheimer disease amyloid plaque pathology. Arch Neurol. 2011; 68:1137–44. 10.1001/archneurol.2011.105 21555603 PMC3154969

[6] Kweon OJ, Youn YC, Lim YK, Lee MK, Kim HR. Clinical utility of serum hepcidin and iron profile measurements in Alzheimer's disease. J Neurol Sci. 2019; 403:85–91. 10.1016/j.jns.2019.06.008 31233974

[7] Petersen RC. Conversion. Neurology. 2006 (Suppl 3); 67:S12–13. 10.1212/wnl.67.9_suppl_3.s12 17101927

[8] Albert MS, DeKosky ST, Dickson D, Dubois B, Feldman HH, Fox NC, Gamst A, Holtzman DM, Jagust WJ, Petersen RC, Snyder PJ, Carrillo MC, Thies B, Phelps CH. The diagnosis of mild cognitive impairment due to Alzheimer's disease: recommendations from the National Institute on Aging-Alzheimer's Association workgroups on diagnostic guidelines for Alzheimer's disease. Alzheimers Dement. 2011; 7:270–79. 10.1016/j.jalz.2011.03.008 21514249 PMC3312027

[9] Roberts R, Knopman DS. Classification and epidemiology of MCI. Clin Geriatr Med. 2013; 29:753–72. 10.1016/j.cger.2013.07.003 24094295 PMC3821397

[10] Petersen RC, Stevens JC, Ganguli M, Tangalos EG, Cummings JL, DeKosky ST. Practice parameter: early detection of dementia: mild cognitive impairment (an evidence-based review). Report of the Quality Standards Subcommittee of the American Academy of Neurology. Neurology. 2001; 56:1133–42. 10.1212/wnl.56.9.1133 11342677

[11] Roundtree IA, Evans ME, Pan T, He C. Dynamic RNA Modifications in Gene Expression Regulation. Cell. 2017; 169:1187–200. 10.1016/j.cell.2017.05.045 28622506 PMC5657247

[12] Boccaletto P, Machnicka MA, Purta E, Piatkowski P, Baginski B, Wirecki TK, de Crécy-Lagard V, Ross R, Limbach PA, Kotter A, Helm M, Bujnicki JM. MODOMICS: a database of RNA modification pathways. 2017 update. Nucleic Acids Res. 2018; 46:D303–07. 10.1093/nar/gkx1030 29106616 PMC5753262

[13] Alarcón CR, Lee H, Goodarzi H, Halberg N, Tavazoie SF. N6-methyladenosine marks primary microRNAs for processing. Nature. 2015; 519:482–85. 10.1038/nature14281 25799998 PMC4475635

[14] Patil DP, Chen CK, Pickering BF, Chow A, Jackson C, Guttman M, Jaffrey SR. m(6)A RNA methylation promotes XIST-mediated transcriptional repression. Nature. 2016; 537:369–73. 10.1038/nature19342 27602518 PMC5509218

[15] Yang Y, Hsu PJ, Chen YS, Yang YG. Dynamic transcriptomic m^6^A decoration: writers, erasers, readers and functions in RNA metabolism. Cell Res. 2018; 28:616–24. 10.1038/s41422-018-0040-8 29789545 PMC5993786

[16] Wang S, Chai P, Jia R, Jia R. Novel insights on m^6^A RNA methylation in tumorigenesis: a double-edged sword. Mol Cancer. 2018; 17:101. 10.1186/s12943-018-0847-4 30031372 PMC6054842

[17] Li Y, Xiao J, Bai J, Tian Y, Qu Y, Chen X, Wang Q, Li X, Zhang Y, Xu J. Molecular characterization and clinical relevance of m^6^A regulators across 33 cancer types. Mol Cancer. 2019; 18:137. 10.1186/s12943-019-1066-3 31521193 PMC6744659

[18] Livneh I, Moshitch-Moshkovitz S, Amariglio N, Rechavi G, Dominissini D. The m^6^A epitranscriptome: transcriptome plasticity in brain development and function. Nat Rev Neurosci. 2020; 21:36–51. 10.1038/s41583-019-0244-z 31804615

[19] Weng YL, Wang X, An R, Cassin J, Vissers C, Liu Y, Liu Y, Xu T, Wang X, Wong SZH, Joseph J, Dore LC, Dong Q, et al. Epitranscriptomic m^6^A Regulation of Axon Regeneration in the Adult Mammalian Nervous System. Neuron. 2018; 97:313–25.e6. 10.1016/j.neuron.2017.12.036 29346752 PMC5777326

[20] Meyer KD, Saletore Y, Zumbo P, Elemento O, Mason CE, Jaffrey SR. Comprehensive analysis of mRNA methylation reveals enrichment in 3' UTRs and near stop codons. Cell. 2012; 149:1635–46. 10.1016/j.cell.2012.05.003 22608085 PMC3383396

[21] Yoon KJ, Ringeling FR, Vissers C, Jacob F, Pokrass M, Jimenez-Cyrus D, Su Y, Kim NS, Zhu Y, Zheng L, Kim S, Wang X, Doré LC, et al. Temporal Control of Mammalian Cortical Neurogenesis by m^6^A Methylation. Cell. 2017; 171:877–89.e17. 10.1016/j.cell.2017.09.003 28965759 PMC5679435

[22] Shi H, Zhang X, Weng YL, Lu Z, Liu Y, Lu Z, Li J, Hao P, Zhang Y, Zhang F, Wu Y, Delgado JY, Su Y, et al. m^6^A facilitates hippocampus-dependent learning and memory through YTHDF1. Nature. 2018; 563:249–53. 10.1038/s41586-018-0666-1 30401835 PMC6226095

[23] Widagdo J, Anggono V. The m6A-epitranscriptomic signature in neurobiology: from neurodevelopment to brain plasticity. J Neurochem. 2018; 147:137–52. 10.1111/jnc.14481 29873074

[24] Zhou H, Wang B, Sun H, Xu X, Wang Y. Epigenetic Regulations in Neural Stem Cells and Neurological Diseases. Stem Cells Int. 2018; 2018:6087143. 10.1155/2018/6087143 29743892 PMC5878882

[25] Yao B, Christian KM, He C, Jin P, Ming GL, Song H. Epigenetic mechanisms in neurogenesis. Nat Rev Neurosci. 2016; 17:537–49. 10.1038/nrn.2016.70 27334043 PMC5610421

[26] Boles NC, Temple S. Epimetronomics: m6A Marks the Tempo of Corticogenesis. Neuron. 2017; 96:718–20. 10.1016/j.neuron.2017.11.002 29144970

[27] Li J, Yang X, Qi Z, Sang Y, Liu Y, Xu B, Liu W, Xu Z, Deng Y. The role of mRNA m^6^A methylation in the nervous system. Cell Biosci. 2019; 9:66. 10.1186/s13578-019-0330-y 31452869 PMC6701067

[28] Lee M, Kim B, Kim VN. Emerging roles of RNA modification: m(6)A and U-tail. Cell. 2014; 158:980–87. 10.1016/j.cell.2014.08.005 25171402

[29] Yue Y, Liu J, He C. RNA N6-methyladenosine methylation in post-transcriptional gene expression regulation. Genes Dev. 2015; 29:1343–55. 10.1101/gad.262766.115 26159994 PMC4511210

[30] Fu Y, Dominissini D, Rechavi G, He C. Gene expression regulation mediated through reversible m^6^A RNA methylation. Nat Rev Genet. 2014; 15:293–306. 10.1038/nrg3724 24662220

[31] Tong J, Cao G, Zhang T, Sefik E, Amezcua Vesely MC, Broughton JP, Zhu S, Li H, Li B, Chen L, Chang HY, Su B, Flavell RA, Li HB. m^6^A mRNA methylation sustains Treg suppressive functions. Cell Res. 2018; 28:253–56. 10.1038/cr.2018.7 29303144 PMC5799823

[32] Qin L, Min S, Shu L, Pan H, Zhong J, Guo J, Sun Q, Yan X, Chen C, Tang B, Xu Q. Genetic analysis of N6-methyladenosine modification genes in Parkinson's disease. Neurobiol Aging. 2020; 93:143.e9–143.e13. 10.1016/j.neurobiolaging.2020.03.018 32371107

[33] Han M, Liu Z, Xu Y, Liu X, Wang D, Li F, Wang Y, Bi J. Abnormality of m6A mRNA Methylation Is Involved in Alzheimer's Disease. Front Neurosci. 2020; 14:98. 10.3389/fnins.2020.00098 32184705 PMC7058666

[34] Arguello AE, DeLiberto AN, Kleiner RE. RNA Chemical Proteomics Reveals the N^6^-Methyladenosine (m^6^A)-Regulated Protein-RNA Interactome. J Am Chem Soc. 2017; 139:17249–52. 10.1021/jacs.7b09213 29140688

[35] Panneerdoss S, Eedunuri VK, Yadav P, Timilsina S, Rajamanickam S, Viswanadhapalli S, Abdelfattah N, Onyeagucha BC, Cui X, Lai Z, Mohammad TA, Gupta YK, Huang TH, et al. Cross-talk among writers, readers, and erasers of m^6^A regulates cancer growth and progression. Sci Adv. 2018; 4:eaar8263. 10.1126/sciadv.aar8263 30306128 PMC6170038

[36] Akichika S, Hirano S, Shichino Y, Suzuki T, Nishimasu H, Ishitani R, Sugita A, Hirose Y, Iwasaki S, Nureki O, Suzuki T. Cap-specific terminal *N*^6^-methylation of RNA by an RNA polymerase II-associated methyltransferase. Science. 2019; 363:eaav0080. 10.1126/science.aav0080 30467178

[37] Ma H, Wang X, Cai J, Dai Q, Natchiar SK, Lv R, Chen K, Lu Z, Chen H, Shi YG, Lan F, Fan J, Klaholz BP, et al. N^6−^Methyladenosine methyltransferase ZCCHC4 mediates ribosomal RNA methylation. Nat Chem Biol. 2019; 15:88–94. 10.1038/s41589-018-0184-3 30531910 PMC6463480

[38] Edgar R, Domrachev M, Lash AE. Gene Expression Omnibus: NCBI gene expression and hybridization array data repository. Nucleic Acids Res. 2002; 30:207–10. 10.1093/nar/30.1.207 11752295 PMC99122

[39] Leek JT, Johnson WE, Parker HS, Jaffe AE, Storey JD. The sva package for removing batch effects and other unwanted variation in high-throughput experiments. Bioinformatics. 2012; 28:882–83. 10.1093/bioinformatics/bts034 22257669 PMC3307112

[40] Alashwal H, El Halaby M, Crouse JJ, Abdalla A, Moustafa AA. The Application of Unsupervised Clustering Methods to Alzheimer's Disease. Front Comput Neurosci. 2019; 13:31. 10.3389/fncom.2019.00031 31178711 PMC6543980

[41] Kiselev VY, Andrews TS, Hemberg M. Challenges in unsupervised clustering of single-cell RNA-seq data. Nat Rev Genet. 2019; 20:273–82. 10.1038/s41576-018-0088-9 30617341

[42] Zhuang J, Lin C, Ye J. m^6^ A RNA methylation regulators contribute to malignant progression in rectal cancer. J Cell Physiol. 2020; 235:6300–06. 10.1002/jcp.29626 32043594

[43] Hartigan J, Wong M. Algorithm AS 136: a K-means clustering algorithm. Appl Stat. 1979; 28:9. 10.2307/2346830

[44] Wilkerson MD, Hayes DN. ConsensusClusterPlus: a class discovery tool with confidence assessments and item tracking. Bioinformatics. 2010; 26:1572–73. 10.1093/bioinformatics/btq170 20427518 PMC2881355

[45] Ritchie ME, Phipson B, Wu D, Hu Y, Law CW, Shi W, Smyth GK. limma powers differential expression analyses for RNA-sequencing and microarray studies. Nucleic Acids Res. 2015; 43:e47. 10.1093/nar/gkv007 25605792 PMC4402510

[46] Hung JH, Yang TH, Hu Z, Weng Z, DeLisi C. Gene set enrichment analysis: performance evaluation and usage guidelines. Brief Bioinform. 2012; 13:281–91. 10.1093/bib/bbr049 21900207 PMC3357488

[47] Song ZY, Chao F, Zhuo Z, Ma Z, Li W, Chen G. Identification of hub genes in prostate cancer using robust rank aggregation and weighted gene co-expression network analysis. Aging (Albany NY). 2019; 11:4736–56. 10.18632/aging.102087 31306099 PMC6660050

[48] Langfelder P, Horvath S. WGCNA: an R package for weighted correlation network analysis. BMC Bioinformatics. 2008; 9:559. 10.1186/1471-2105-9-559 19114008 PMC2631488

[49] Zhang B, Wu Q, Li B, Wang D, Wang L, Zhou YL. m^6^A regulator-mediated methylation modification patterns and tumor microenvironment infiltration characterization in gastric cancer. Mol Cancer. 2020; 19:53. 10.1186/s12943-020-01170-0 32164750 PMC7066851

[50] Horvath S, Zhang B, Carlson M, Lu KV, Zhu S, Felciano RM, Laurance MF, Zhao W, Qi S, Chen Z, Lee Y, Scheck AC, Liau LM, et al. Analysis of oncogenic signaling networks in glioblastoma identifies ASPM as a molecular target. Proc Natl Acad Sci U S A. 2006; 103:17402–07. 10.1073/pnas.0608396103 17090670 PMC1635024

[51] Corder EH, Saunders AM, Strittmatter WJ, Schmechel DE, Gaskell PC, Small GW, Roses AD, Haines JL, Pericak-Vance MA. Gene dose of apolipoprotein E type 4 allele and the risk of Alzheimer's disease in late onset families. Science. 1993; 261:921–23. 10.1126/science.8346443 8346443

[52] Foraker J, Millard SP, Leong L, Thomson Z, Chen S, Keene CD, Bekris LM, Yu CE. The APOE Gene is Differentially Methylated in Alzheimer's Disease. J Alzheimers Dis. 2015; 48:745–55. 10.3233/JAD-143060 26402071 PMC6469491

[53] Tannorella P, Stoccoro A, Tognoni G, Petrozzi L, Salluzzo MG, Ragalmuto A, Siciliano G, Haslberger A, Bosco P, Bonuccelli U, Migliore L, Coppedè F. Methylation analysis of multiple genes in blood DNA of Alzheimer's disease and healthy individuals. Neurosci Lett. 2015; 600:143–47. 10.1016/j.neulet.2015.06.009 26079324

[54] Li L, Zang L, Zhang F, Chen J, Shen H, Shu L, Liang F, Feng C, Chen D, Tao H, Xu T, Li Z, Kang Y, et al. Fat mass and obesity-associated (FTO) protein regulates adult neurogenesis. Hum Mol Genet. 2017; 26:2398–411. 10.1093/hmg/ddx128 28398475 PMC6192412

[55] Hess ME, Hess S, Meyer KD, Verhagen LA, Koch L, Brönneke HS, Dietrich MO, Jordan SD, Saletore Y, Elemento O, Belgardt BF, Franz T, Horvath TL, et al. The fat mass and obesity associated gene (Fto) regulates activity of the dopaminergic midbrain circuitry. Nat Neurosci. 2013; 16:1042–48. 10.1038/nn.3449 23817550

[56] Widagdo J, Zhao QY, Kempen MJ, Tan MC, Ratnu VS, Wei W, Leighton L, Spadaro PA, Edson J, Anggono V, Bredy TW. Experience-Dependent Accumulation of N6-Methyladenosine in the Prefrontal Cortex Is Associated with Memory Processes in Mice. J Neurosci. 2016; 36:6771–77. 10.1523/JNEUROSCI.4053-15.2016 27335407 PMC4916251

[57] Walters BJ, Mercaldo V, Gillon CJ, Yip M, Neve RL, Boyce FM, Frankland PW, Josselyn SA. The Role of The RNA Demethylase FTO (Fat Mass and Obesity-Associated) and mRNA Methylation in Hippocampal Memory Formation. Neuropsychopharmacology. 2017; 42:1502–10. 10.1038/npp.2017.31 28205605 PMC5436121

[58] Zhang Z, Wang M, Xie D, Huang Z, Zhang L, Yang Y, Ma D, Li W, Zhou Q, Yang YG, Wang XJ. METTL3-mediated N^6^-methyladenosine mRNA modification enhances long-term memory consolidation. Cell Res. 2018; 28:1050–61. 10.1038/s41422-018-0092-9 30297870 PMC6218447

[59] Koranda JL, Dore L, Shi H, Patel MJ, Vaasjo LO, Rao MN, Chen K, Lu Z, Yi Y, Chi W, He C, Zhuang X. Mettl14 Is Essential for Epitranscriptomic Regulation of Striatal Function and Learning. Neuron. 2018; 99:283–92.e5. 10.1016/j.neuron.2018.06.007 30056831 PMC6082022

[60] Xu H, Dzhashiashvili Y, Shah A, Kunjamma RB, Weng YL, Elbaz B, Fei Q, Jones JS, Li YI, Zhuang X, Ming GL, He C, Popko B. m^6^A mRNA Methylation Is Essential for Oligodendrocyte Maturation and CNS Myelination. Neuron. 2020; 105:293–309.e5. 10.1016/j.neuron.2019.12.013 31901304 PMC7137581

[61] Wu R, Li A, Sun B, Sun JG, Zhang J, Zhang T, Chen Y, Xiao Y, Gao Y, Zhang Q, Ma J, Yang X, Liao Y, et al. A novel m^6^A reader Prrc2a controls oligodendroglial specification and myelination. Cell Res. 2019; 29:23–41. 10.1038/s41422-018-0113-8 30514900 PMC6318280

[62] Du B, Li H, Zheng H, Fan C, Liang M, Lian Y, Wei Z, Zhang Y, Bi X. Minocycline Ameliorates Depressive-Like Behavior and Demyelination Induced by Transient Global Cerebral Ischemia by Inhibiting Microglial Activation. Front Pharmacol. 2019; 10:1247. 10.3389/fphar.2019.01247 31695615 PMC6817504

[63] Du B, Liang M, Zheng H, Fan C, Zhang H, Lu X, Du Z, Lian Y, Zhang Y, Bi X. Anti-mouse CX3CR1 Antibody Alleviates Cognitive Impairment, Neuronal Loss and Myelin Deficits in an Animal Model of Brain Ischemia. Neuroscience. 2020; 438:169–81. 10.1016/j.neuroscience.2020.05.011 32417340

[64] Kelly J, Moyeed R, Carroll C, Luo S, Li X. Genetic networks in Parkinson's and Alzheimer's disease. Aging (Albany NY). 2020; 12:5221–43. 10.18632/aging.102943 32205467 PMC7138567

[65] Papassotiropoulos A, Gerhards C, Heck A, Ackermann S, Aerni A, Schicktanz N, Auschra B, Demougin P, Mumme E, Elbert T, Ertl V, Gschwind L, Hanser E, et al. Human genome-guided identification of memory-modulating drugs. Proc Natl Acad Sci U S A. 2013; 110:E4369–74. 10.1073/pnas.1314478110 24145423 PMC3831994

[66] Shin YK, Cong WN, Cai H, Kim W, Maudsley S, Egan JM, Martin B. Age-related changes in mouse taste bud morphology, hormone expression, and taste responsivity. J Gerontol A Biol Sci Med Sci. 2012; 67:336–44. 10.1093/gerona/glr192 22056740 PMC3410661

[67] Martin B, Maudsley S, White CM, Egan JM. Hormones in the naso-oropharynx: endocrine modulation of taste and smell. Trends Endocrinol Metab. 2009; 20:163–70. 10.1016/j.tem.2009.01.006 19359194 PMC2732121

[68] Martin B, Wang R, Cong WN, Daimon CM, Wu WW, Ni B, Becker KG, Lehrmann E, Wood WH 3rd, Zhang Y, Etienne H, van Gastel J, Azmi A, et al. Altered learning, memory, and social behavior in type 1 taste receptor subunit 3 knock-out mice are associated with neuronal dysfunction. J Biol Chem. 2017; 292:11508–30. 10.1074/jbc.M116.773820 28522608 PMC5500814

[69] Woolley JD, Gorno-Tempini ML, Seeley WW, Rankin K, Lee SS, Matthews BR, Miller BL. Binge eating is associated with right orbitofrontal-insular-striatal atrophy in frontotemporal dementia. Neurology. 2007; 69:1424–33. 10.1212/01.wnl.0000277461.06713.23 17909155

[70] Ahmed RM, Irish M, Kam J, van Keizerswaard J, Bartley L, Samaras K, Hodges JR, Piguet O. Quantifying the eating abnormalities in frontotemporal dementia. JAMA Neurol. 2014; 71:1540–46. 10.1001/jamaneurol.2014.1931 25329012

[71] Mendez MF, Licht EA, Shapira JS. Changes in dietary or eating behavior in frontotemporal dementia versus Alzheimer's disease. Am J Alzheimers Dis Other Demen. 2008; 23:280–85. 10.1177/1533317507313140 18198236 PMC10846021

[72] Ren X, Zhou L, Terwilliger R, Newton SS, de Araujo IE. Sweet taste signaling functions as a hypothalamic glucose sensor. Front Integr Neurosci. 2009; 3:12. 10.3389/neuro.07.012.2009 19587847 PMC2706652

[73] Talevi A, Enrique AV, Bruno-Blanch LE. Anticonvulsant activity of artificial sweeteners: a structural link between sweet-taste receptor T1R3 and brain glutamate receptors. Bioorg Med Chem Lett. 2012; 22:4072–74. 10.1016/j.bmcl.2012.04.076 22579423

[74] Ménard C, Quirion R. Group 1 metabotropic glutamate receptor function and its regulation of learning and memory in the aging brain. Front Pharmacol. 2012; 3:182. 10.3389/fphar.2012.00182 23091460 PMC3469824

[75] Heinrichs V, Hackl W, Lührmann R. Direct binding of small nuclear ribonucleoprotein G to the Sm site of small nuclear RNA. Ultraviolet light cross-linking of protein G to the AAU stretch within the Sm site (AAUUUGUGG) of U1 small nuclear ribonucleoprotein reconstituted *in vitro*. J Mol Biol. 1992; 227:15–28. 10.1016/0022-2836(92)90678-d 1387914

[76] Tao Y, Han Y, Yu L, Wang Q, Leng SX, Zhang H. The Predicted Key Molecules, Functions, and Pathways That Bridge Mild Cognitive Impairment (MCI) and Alzheimer's Disease (AD). Front Neurol. 2020; 11:233. 10.3389/fneur.2020.00233 32308643 PMC7145962

[77] Azam S, Hou S, Zhu B, Wang W, Hao T, Bu X, Khan M, Lei H. Nuclear retention element recruits U1 snRNP components to restrain spliced lncRNAs in the nucleus. RNA Biol. 2019; 16:1001–09. 10.1080/15476286.2019.1620061 31107149 PMC6602561

[78] Feng Y, Wang X. Systematic analysis of microarray datasets to identify Parkinson's disease-associated pathways and genes. Mol Med Rep. 2017; 15:1252–62. 10.3892/mmr.2017.6124 28098893 PMC5367356

[79] Liu CC, Liu CC, Kanekiyo T, Xu H, Bu G. Apolipoprotein E and Alzheimer disease: risk, mechanisms and therapy. Nat Rev Neurol. 2013; 9:106–18. 10.1038/nrneurol.2012.263 23296339 PMC3726719

[80] Lee EG, Tulloch J, Chen S, Leong L, Saxton AD, Kraemer B, Darvas M, Keene CD, Shutes-David A, Todd K, Millard S, Yu CE. Redefining transcriptional regulation of the APOE gene and its association with Alzheimer's disease. PLoS One. 2020; 15:e0227667. 10.1371/journal.pone.0227667 31978088 PMC6980611

[81] Keller L, Xu W, Wang HX, Winblad B, Fratiglioni L, Graff C. The obesity related gene, FTO, interacts with APOE, and is associated with Alzheimer's disease risk: a prospective cohort study. J Alzheimers Dis. 2011; 23:461–69. 10.3233/JAD-2010-101068 21098976

[82] Ling IF, Estus S. Role of SFRS13A in low-density lipoprotein receptor splicing. Hum Mutat. 2010; 31:702–09. 10.1002/humu.21244 20232416 PMC3184548

[83] Arun V, Wiley JC, Kaur H, Kaplan DR, Guha A. A novel neurofibromin (NF1) interaction with the leucine-rich pentatricopeptide repeat motif-containing protein links neurofibromatosis type 1 and the French Canadian variant of Leigh's syndrome in a common molecular complex. J Neurosci Res. 2013; 91:494–505. 10.1002/jnr.23189 23361976

