# Supplementary material for: N6-methyladenosine (m6A) modification and its clinical relevance in cognitive dysfunctions
Source: Aging (Albany NY). 2021 Aug 30;13(16):20716–37. doi: 10.18632/aging.203457 (PMC8436914; doi:10.18632/aging.203457)
Supplement: Supplementary Figures [file aging-13-203457-s001.pdf]

SUPPLEMENTARY FIGURES

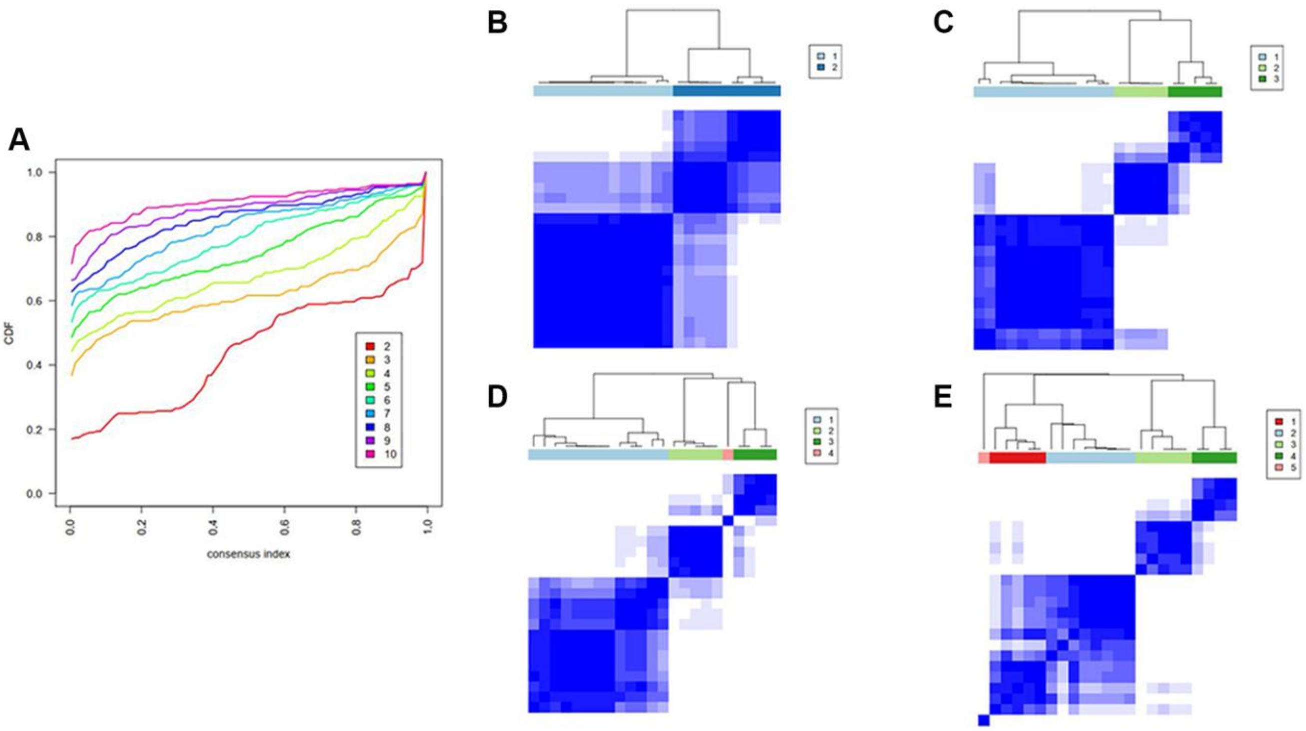

Supplementary Figure 1. The unsupervised cluster based on ConsensusClusterPlus in R package. (**A**) Empirical cumulative distribution function (CDF) plots display consensus distributions for each *k*. (**B**–**E**) Consensus matrices of dementia cohort for *k* = 2 – 5.

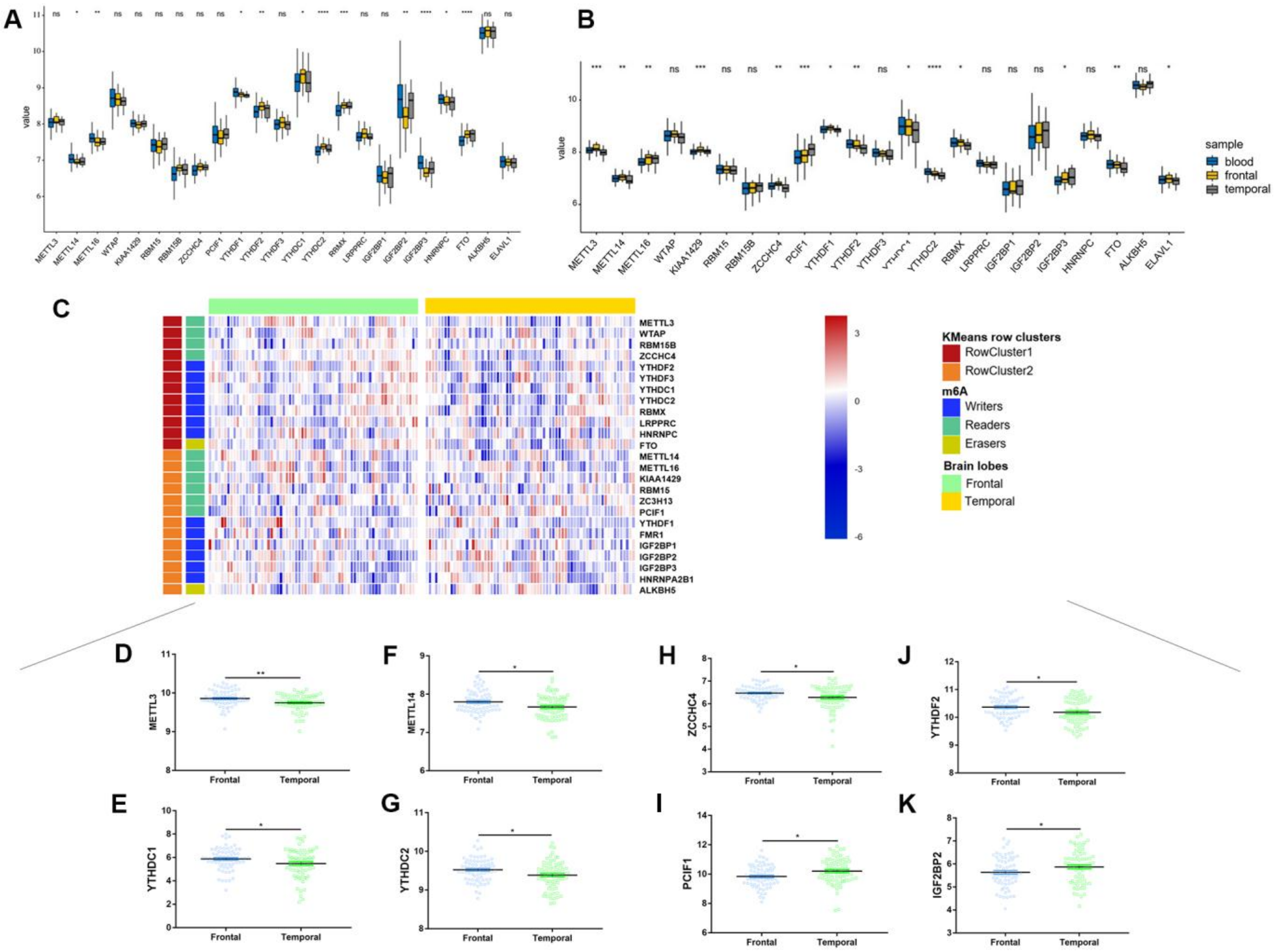

Supplementary Figure 2. Mutation frequency distribution of m6A regulators across different samples or brain regions. (**A**–**B**) The expression profile of 26 m6A regulators in CTL and AD groups. (**C**) Unsupervised clustering of 26 m6A regulators in GSE122063, annotated according to brain regions. Red represented high expression of regulators and blue represented low expression. (**D**–**K**) Box plots showing representative expression differences between frontal and temporal lobes. $^{*}p < 0.05$, $^{**}p < 0.01$, $^{***}p < 0.001$, $^{****}p < 0.0001$, ns: no significance.

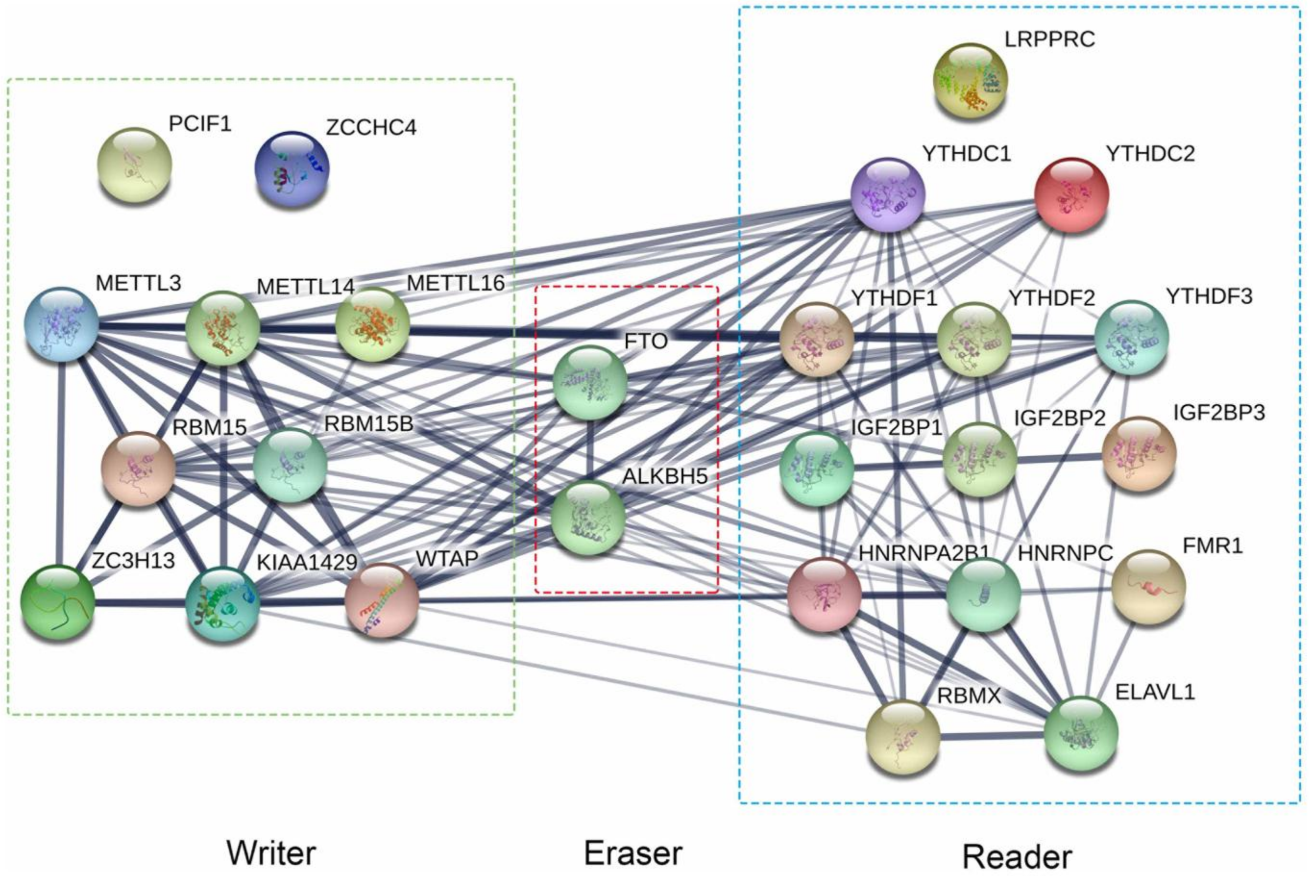

Supplementary Figure 3. Overview of the PPI network among the 26 m6A methylation regulators constructed using STRING.

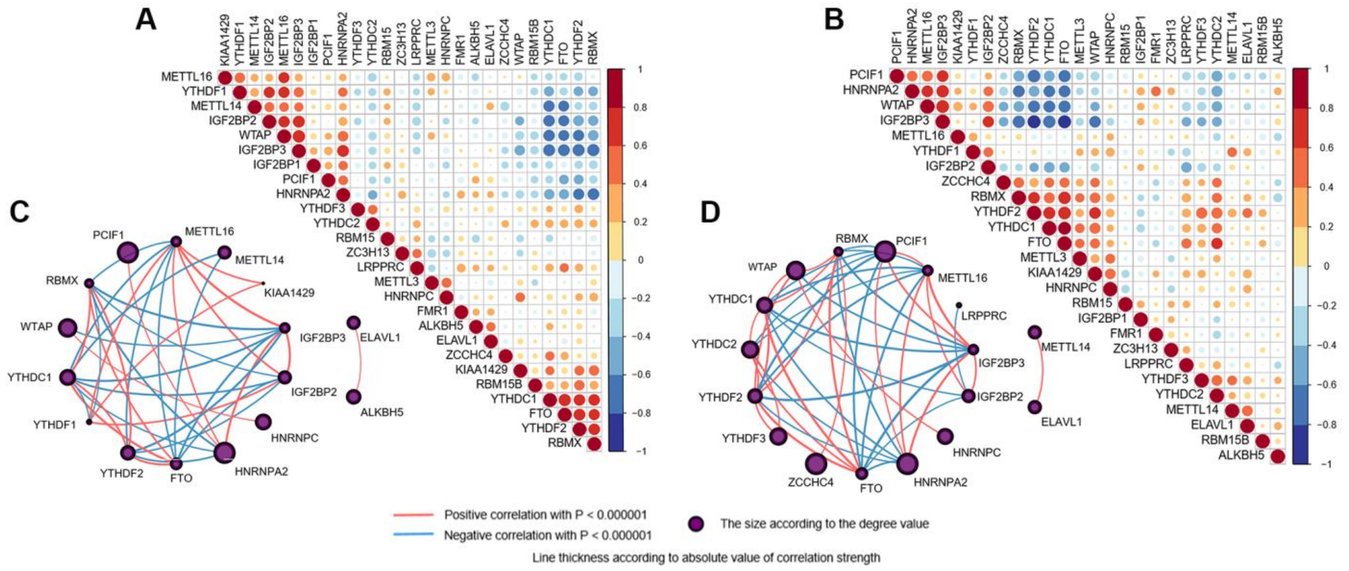

Supplementary Figure 4. Interaction among m6A RNA methylation regulators according to brain regions. (**A**, **B**) Spearman correlation analysis of the 26 m6A methylation regulators in frontal (**A**) and temporal (**B**) lobes. Positive correlation was marked with red and negative correlation with blue. (**C**, **D**) The interaction between the 26 m6A methylation regulators by constructing PPI network in frontal (**C**) and temporal (**D**) samples, respectively. The circle size was determined by the degree value. The lines linking regulators showed their interactions, and thickness showed the correlation strength between regulators. Positive correlation was marked with red and negative correlation with blue.

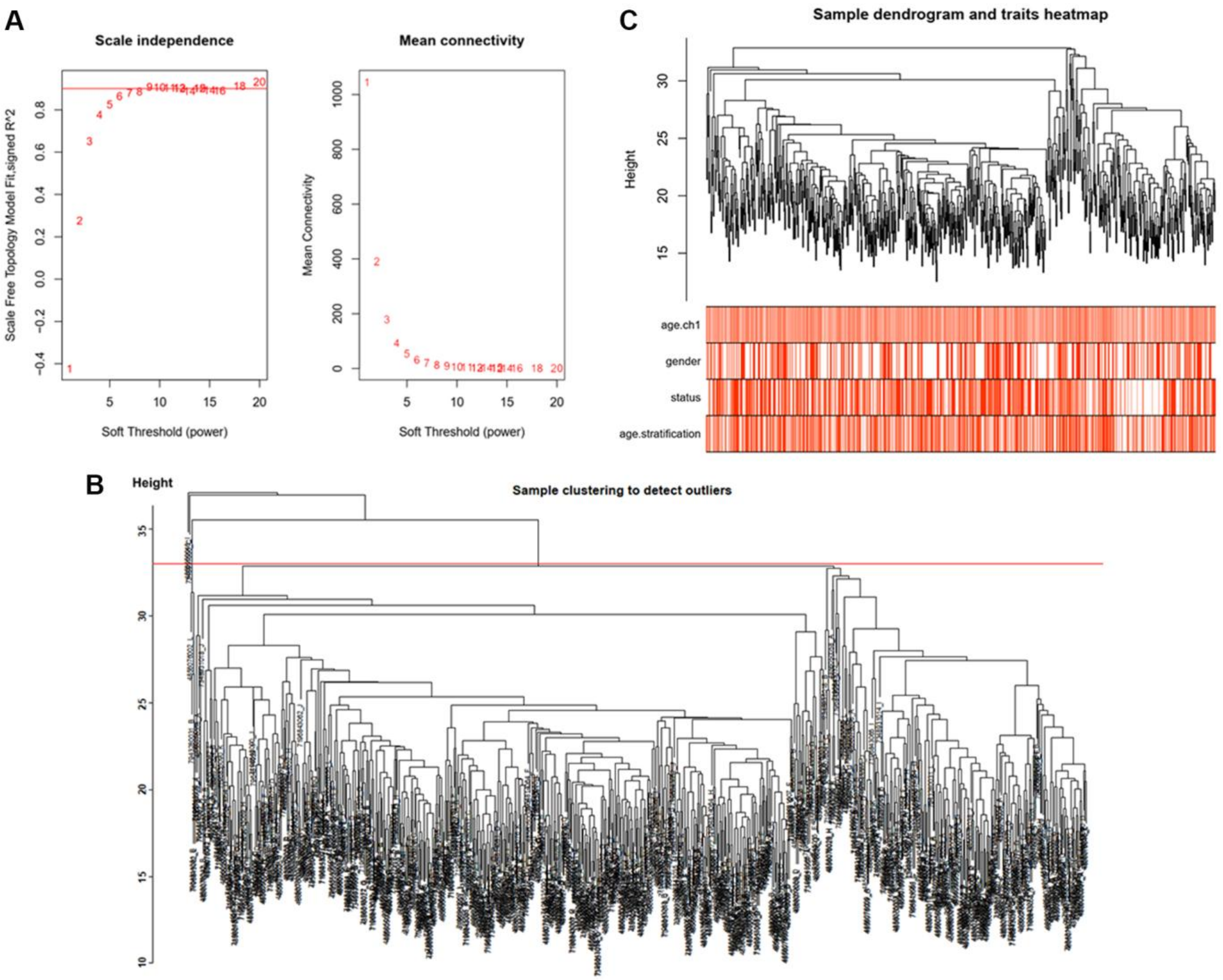

Supplementary Figure 5. Preparation before identification of WGCNA key modules. (**A**) Analysis of network topology for different soft-thresholding powers. The left panel shows the dependence of the scale-free fit index (y-axis) on the soft-thresholding power (x-axis). The right panel displays the influence of soft-thresholding power (x-axis) on the mean connectivity (degree, y-axis). (**B**) Sample clustering to detect outliers after setting height = 33. (**C**) Clustering dendrograms of genes based on clinical traits. Color intensity varies positively with age/ age stratification, gender and cognitive function.
