# Supplementary material for: N6-methyladenosine (m6A) modification and its clinical relevance in cognitive dysfunctions
Source: Aging (Albany NY). 2021 Aug 30;13(16):20716–37. doi: 10.18632/aging.203457 (PMC8436914; doi:10.18632/aging.203457)
Supplement: Supplementary Tables [file aging-13-203457-s002.pdf]

SUPPLEMENTARY TABLES

Supplementary Table 1. Age stratification in specific cognitive function groups.

Groups	Total *n* (%)	≤70 years old *n* (%)	71-79 years old *n* (%)	≥80 years old *n* (%)	χ^2 Test	
					χ^2	*P* Value
CTL	238 (33.5)	68 (43.1)	127 (37.1)	43 (20.5)*	26.2	<0.001
MCI	189 (26.6)	37 (23.4)	90 (26.2)	62 (29.5)		
AD	284 (39.9)	53 (33.5)	126 (36.7)	105 (50.0)*		
Total	711	158 (22.2)	343 (48.2)	210 (29.5)		

*$P < 0.05$ compared to other age groups.

Supplementary Table 2-1. Prediction of the prevalence from CTL to MCI.

	B	SE	*P*	OR	95% CI
Age	0.054	0.017	0.001	1.056	(1.022, 1.091)
WTAP	–1.699	0.439	<0.001	0.183	(0.077, 0.433)
ZCCHC4	–2.814	1.523	0.065	0.060	(0.003, 1.186)
HNRNPC	–2.234	0.658	0.001	0.107	(0.029, 0.389)
Constant	47.361	11.777	<0.001		

Supplementary Table 2-2. Prediction of the prevalence from CTL to AD.

	B	SE	*P*	OR	95% CI
Age	0.054	0.015	<0.001	1.055	(1.025, 1.087)
METTL3	1.527	0.699	0.029	4.603	(1.170, 18.118)
METTL14	–2.079	1.067	0.051	0.125	(0.015, 1.013)
WTAP	–1.198	0.494	0.015	0.302	(0.115, 0.795)
YTHDF3	2.084	0.698	0.003	8.033	(2.047, 31.523)
YTHDC1	–1.173	0.493	0.017	0.309	(0.118, 0.813)
IGF2BP2	–0.465	0.174	0.007	0.628	(0.447, 0.883)
HNRNPC	–1.432	0.599	0.017	0.239	(0.074, 0.772)
FTO	–2.409	0.949	0.011	0.090	(0.014, 0.578)
ALKBH5	1.625	0.550	0.003	5.079	(1.728, 14.931)
Constant	19.863	13.301	0.135		

Supplementary Table 2-3. Prediction of the prevalence from MCI to AD.

	B	SE	P	OR	95% CI
Gender	0.399	0.202	0.048	1.490	(1.003, 2.213)
METTL3	1.944	0.698	0.005	6.984	(1.778, 27.432)
WTAP	0.964	0.496	0.052	2.621	(0.991, 6.935)
RBM15	1.473	0.623	0.018	4.361	(1.287, 14.783)
YTHDF1	–2.235	0.796	0.005	0.107	(0.022, 0.50)
YTHDC1	–0.957	0.489	0.051	0.384	(0.147, 1.002)
LRPPRC	–1.945	0.766	0.011	0.143	(0.032, 0.642)
Constant	7.798	9.232	0.398		
